# Cyclization Strategies in Carbonyl–Olefin Metathesis: An Up-to-Date Review

**DOI:** 10.3390/molecules29204861

**Published:** 2024-10-14

**Authors:** Xiaoke Zhang

**Affiliations:** 1 School of Preclinical Medicine, Zunyi Medical University, Zunyi 563006, China; xiaokezhang53@163.com; 2 Institute of Life Sciences, Zunyi Medical University, Zunyi 563006, China

**Keywords:** cyclization, carbonyl–olefin metathesis, monocyclic compounds, bicyclic compounds, polycyclic compounds

## Abstract

The metathesis reaction between carbonyl compounds and olefins has emerged as a potent strategy for facilitating swift functional group interconversion and the construction of intricate organic structures through the creation of novel carbon–carbon double bonds. To date, significant progress has been made in carbonyl–olefin metathesis reactions, where oxetane, pyrazolidine, 1,3-diol, and metal alkylidene have been proved to be key intermediates. Recently, several reviews have been disclosed, focusing on distinct catalytic approaches for achieving carbonyl–olefin metathesis. However, the summarization of cyclization strategies for constructing aromatic heterocyclic frameworks through carbonyl–olefin metathesis reactions has rarely been reported. Consequently, we present an up-to-date review of the cyclization strategies in carbonyl–olefin metathesis, categorizing them into three main groups: the formation of monocyclic compounds, bicyclic compounds, and polycyclic compounds. This review delves into the underlying mechanism, scope, and applications, offering a comprehensive perspective on the current strength and the limitation of this field.

## 1. Introduction

**Figure 1:**
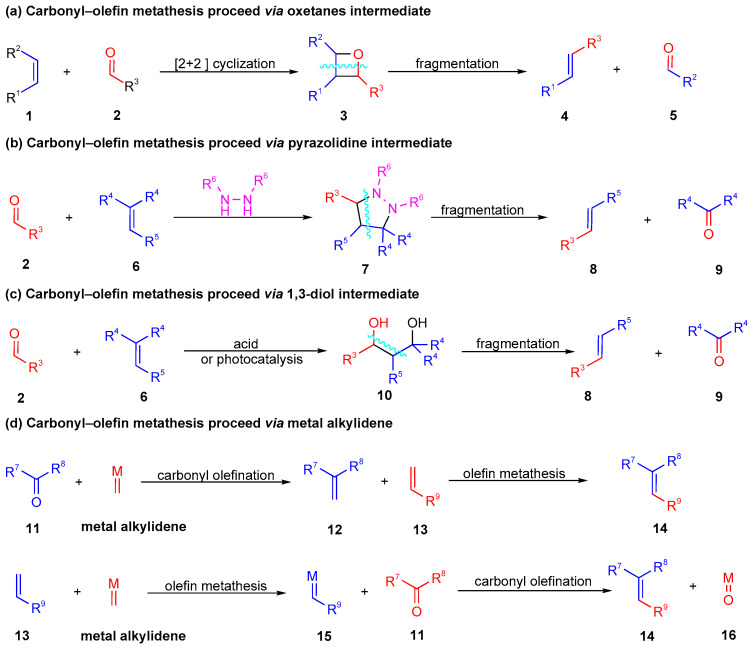
Strategies for carbonyl–olefin metathesis.

Since the groundbreaking awarding of the Nobel Prize in Chemistry to olefin me-tathesis and the palladium-catalyzed cross-coupling reaction in 2005 and 2010, the field of carbon–carbon bond formation has witnessed remarkable advancements [1]. Notably, ole-fin metathesis has found widespread applications across pharmaceutical, agricultural, materials science, and petroleum industries [2,3,4,5,6,7,8,9,10,11]. This includes the synthesis of intricate natural products such as ingenol, (–)-mucocin, coleophomones B, ciguatoxin CTX3C, and amphidinolide A, achieved through ring-closing metathesis macrocyclization [12,13,14,15,16]. Additionally, cross-metathesis degradation and ring-opening metathesis polymerization techniques hold promise for functional rubber synthesis [17,18,19,20,21,22]. In contrast, the carbonyl–olefin metathesis strategy has lagged behind, hindered by the need for stoichiometric amounts of transition metal reagents, photochemical promotion, or a substrate prone to cationic cyclization. Despite these challenges, innovative carbonyl–olefin metathesis protocols have emerged as valuable complements to olefin metathesis, enabling the synthesis of conjugate polymers, biologically active molecules, and complex natural products [23,24,25,26,27,28,29,30,31]. For instance, chain alkenes and enals can be generated through an intermolecular car-bonyl–olefin exchange [32,33,34,35,36,37], while intramolecular reactions facilitate the synthesis of conjugate polymers and natural products [38,39,40,41]. These diverse methodologies can be categorized based on their reaction intermediates: (a) carbonyl–olefin metathesis proceeds via oxetane intermediates (Figure 1a), involving the Paternò–Büchi reaction between alkene and carbonyl under UV light, leading to oxetanes that fragment under acidic condition or high temperature to yield a new alkene and carbonyl [42,43]; (b) carbonyl–olefin metathesis proceeds via pyrazolidine intermediates (Figure 1b), achieved through the 1,3-dipolar cycloaddition of an alkene with azomethine imine generated from hydrazine and an aldehyde, followed by cycloreversion to produce new olefin and ketone [26]; (c) carbonyl–olefin metathesis proceeds via 1,3-diol intermediates (Figure 1c), where the intermolecular addition of a C–O double bond to an alkene-derived radical, prepared via a photoinitiated hole catalytic cycle, forms a reactive 1,3-diol that undergoes Grob fragmentation to yield olefin and ketone [44]; and (d) carbonyl–olefin metathesis proceeds via a metal alkylidene intermediate (Figure 1d), where the olefination of a carbonyl is facilitated by metal alkylidene, with subsequent olefin–olefin metathesis or a direct carbonyl olefination reaction producing the desired product [45,46]. While several recent reviews have focused on different catalytic approaches for the carbonyl–olefin metathesis proceeds via the intermediate oxetane metathesis reaction and complex molecule synthesis [26,27,28,30], a comprehensive overview of the cyclization strategies within this field remains limited.

**Figure 2:**
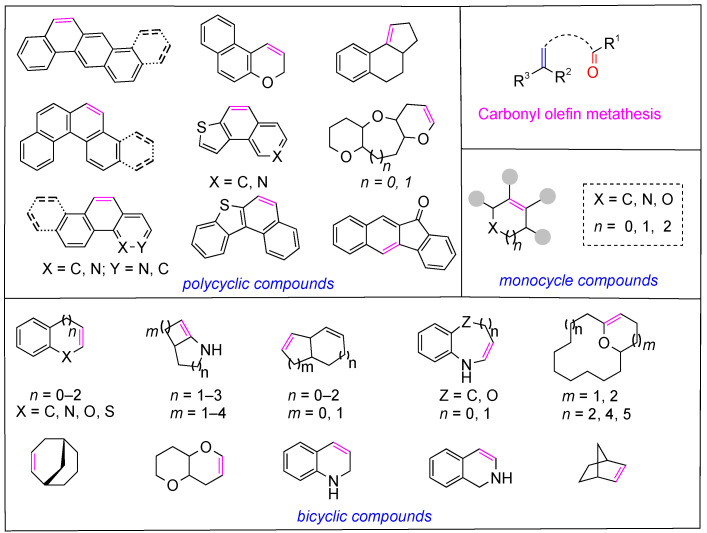
The classification of carbonyl–olefin metathesis.

In this up-to-date review, we summarize the cyclization strategies in carbonyl–olefin metathesis, dividing them into three categories (Figure 2): (1) the formation of monocyclic compounds; (2) the formation of bicyclic compounds; and (3) the formation of polycyclic compounds.

## 2. The Formation of Monocyclic Compounds

This section includes two major parts: (1) a carbonyl–olefin metathesis cyclization reaction catalyzed by metal; and (2) a carbonyl–olefin metathesis cyclization reaction catalyzed by metal-free condition.

### 2.1. Carbonyl–Olefin Metathesis Cyclization Reaction Catalyzed by Metal

**Scheme 1:**
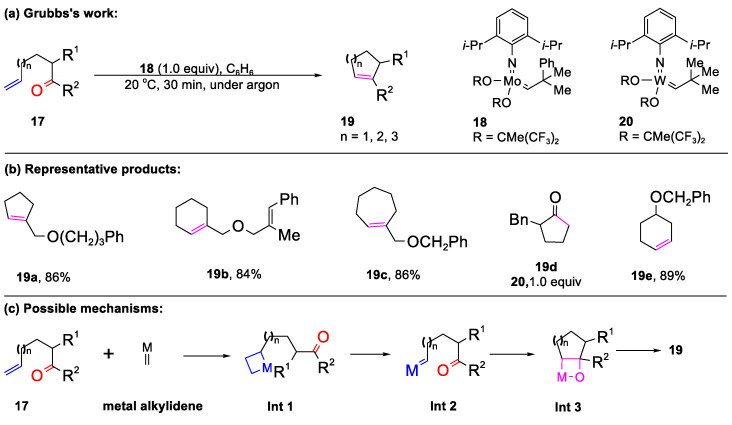
The synthesis of cycloalkenes via carbonyl–olefin metathesis reaction.

The earliest reported instance of a carbonyl–olefin metathesis cyclization reaction, catalyzed by a metal to yield five-membered ring scaffolds, dates back to Grubbs’ work in 1993 [47]. When an olefinic ketone **17** served as the substrate, intramolecular cyclization between the olefin and carbonyl functional group occurred, with the aid of stoichiometric amounts of molybdenum alkylidene **18**, yielding the cycloalkene **19** using benzene as a solvent (Scheme 1). This approach showcased a versatile substrate scope, enabling the synthesis of diverse unsaturated five-, six-, and seven-membered rings (**19a**–**19c**) in good yields. Notably, the carbonyl–olefin metathesis of olefinic ketone **17**, featuring *α*-oxygenation, leads to the formation of cyclic ketone **19d** via the isomerization of the cyclic enol ether intermediate, facilitated by **20** (1.0 equiv). Interestingly, **19e** bearing an ester functional group could be obtained from functionalized diene as the starting material, utilizing 2 mol% of **18**. Drawing from previous studies [48,49], the author hypothesized that the olefin metathesis between olefinic ketone **17** and **18** generated a new alkylidene intermediate, **Int 2,** containing a carbonyl group, which subsequently underwent intramolecular carbonyl olefination, mediated by metal quaternary ring intermediate **Int 3**, to produce cycloalkene **19**.

**Scheme 2:**
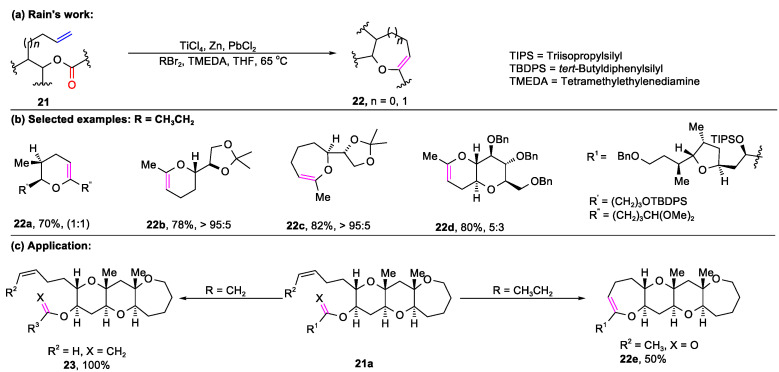
Olefinic ester ring-closing metathesis: using a reduced titanium akylidene.

In 2007, Rainier’s team elaborated on the exceptional and unprecedented reactivity demonstrated by an in situ-produced reduced titanium ethylidene reagent, which was found to interact with carbonyl functional groups in a unique manner (Scheme 2) [50]. This interaction led to the formation of cyclic enol ether **22** via a ring-closing metathesis reaction, marking a significant advancement in the field. This approach efficiently pro-vided the desired products **22a**–**22d** in moderate yields through a two-step metathesis sequence. Additionally, the gambieric acid A derivative **22e** was achieved with a 50% yield via this transformation (Scheme 2c). Specifically, when using dibromomethane as the alkylidene source, the resulting titanium methylidene reagent exclusively produced acyclic enol ether **23**.

**Scheme 3:**
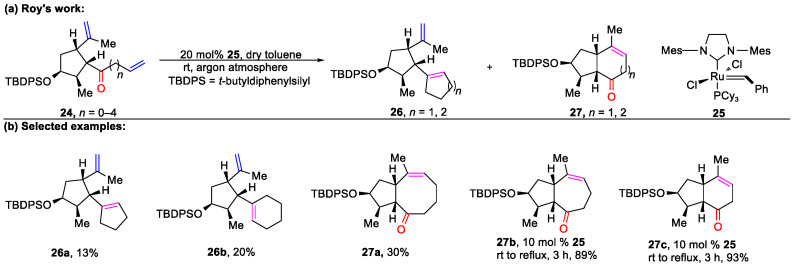
Diversity-oriented synthesis of carbocyclic.

Inspired by previous work, Roy and colleagues developed a Grubbs–II **25**-catalyzed ring-closing carbonyl–olefin metathesis and ring-closing olefin metathesis protocol for the synthesis of unsaturated carbocyclic compounds **26** and **27** (Scheme 3) [51]. When n = 3 in **24**, the carbonyl–olefin metathesis product **26a** and olefin metathesis product **27a** were observed. When n = 4 in **24**, only the ring-closing carbonyl–olefin metathesis product **26b** was formed in a moderate yield of 20%. In addition, for **24** with n < 4, the olefin metathesis proceeded efficiently using 10 mol% Grubbs-II **25** under reflux conditions, providing a series of bicyclic frameworks, including five–seven (**27b**) and five–six (**27c**), in high yields. Further experimental studies have revealed that increasing the loading of **25** favored the ring-closing carbonyl–olefin metathesis of **26**.

**Scheme 4:**
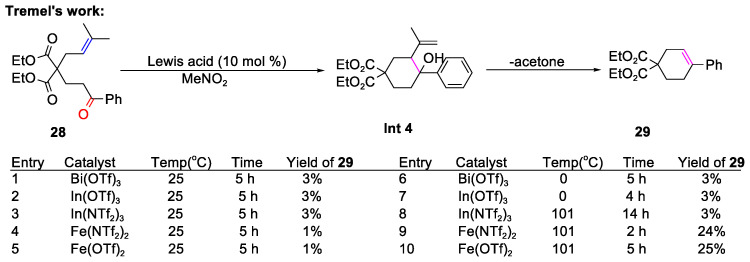
Catalytic intramolecular carbonyl–olefin reaction.

Later, Duñach’s group reported a Lewis acid-catalyzed intramolecular carbonyl–olefin ring-closing process of phenyl ketone **28**, giving cyclohexene **29** (Scheme 4) [52]. Further ESI-MS analysis revealed that the dehydration of the tertiary alcohol **Int 4** served as the primary side reaction, contributing to the reduced yield of **29**. Conditional screening experiments have demonstrated that Fe(NTf_2_)_2_ has potential for promoting the metathesis of carbonyl olefin.

**Scheme 5:**
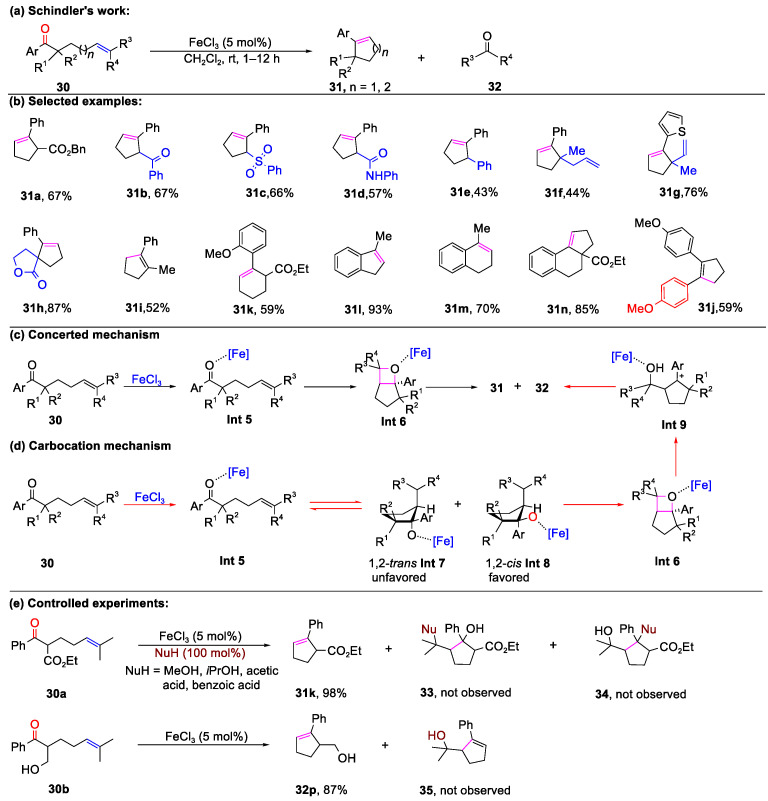
Iron(III)-catalyzed carbonyl–olefin metathesis.

Despite the well-established metal-catalyzed carbonyl olefin metathesis reaction, its application has been hindered by the necessity of stoichiometric transition metals or harsh reaction conditions [24,53]. In 2016, Schindler’s group overcame this limitation by demonstrating a catalytic FeCl_3_-mediated carbonyl–olefin ring-closing metathesis reaction that efficiently prepared unsaturated carbocyclic olefin in moderate to excellent yields (Scheme 5) [54]. This process used aromatic *β*-ketoester **30** with a pendant isoprenyl group as a substrate, under mild conditions, facilitating advances in olefin metathesis research. The reaction exhibited high functional group tolerance and a wide substrate scope, ena-bling the conversion of variously substituted aromatic *β*-ketoesters into the cyclopentene product. Notably, various *β*-substitutions, including a benzyl ester, ketone, sulfonyl, and amide, were compatible, providing cyclopentenes **31a**–**31h** with up to 87% efficiency. For methyl or *p*-phenyl methoxy substitution, only thermodynamically stable product **31i** was observed. Remarkably, spirocyclic motif **31h**, six-membered carbocycle **31k**, indene **31l**, 1,2-dihydronaphthalene **31m**, and tricyclic **31n** could also be efficiently synthesized through this carbonyl–olefin metathesis. Based on their findings, Schindler and colleagues proposed two potential mechanisms: a concerted mechanism involving [2+2] cycloaddition and cycloreversion (Scheme 5c) and a carbocation mechanism featuring nucleophilic attack and carbocation trapping (Scheme 5d). Further experiments revealed the absence of a carbocation intermediate, supporting the concerted mechanism (Scheme 5e).

**Scheme 6:**
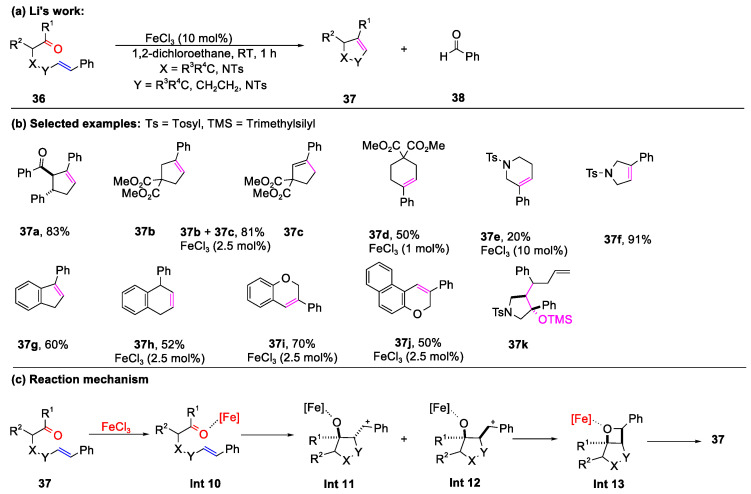
FeCl_3_-catalyzed ring-closing carbonyl–olefin metathesis.

Concurrently, Li and co-workers disclosed an efficient FeCl_3_-catalyzed ring-closing carbonyl–olefin metathesis using 1,2-dichloroethane (DCE) as a solvent (Scheme 6) [55]. This method could provide cyclopentene **37a**, cyclohexene **37d**, tetrahydropyridine **37e**, 2,5-dihydro-pyrrole **37f**, indene **37g**, 1,4-dihydronaphthalene **37h**, and 2,5-dihydro-1*H*-pyrrole **37i**, with good yields using 1–20 mol% FeCl_3_. Notably, allyl trimethyl silane facilitated key oxetane intermediate formation and benzaldehyde scavenging, which are crucial for the synthesis of five-membered nitrogen heterocycle **37f**. The presence of minor side product **37k** led to the proposal of a stepwise mechanism (Scheme 6c) [56,57], involving electrophilic cyclization, [2+2] cycloreversion, and FeCl_3_ regeneration.

**Scheme 7:**
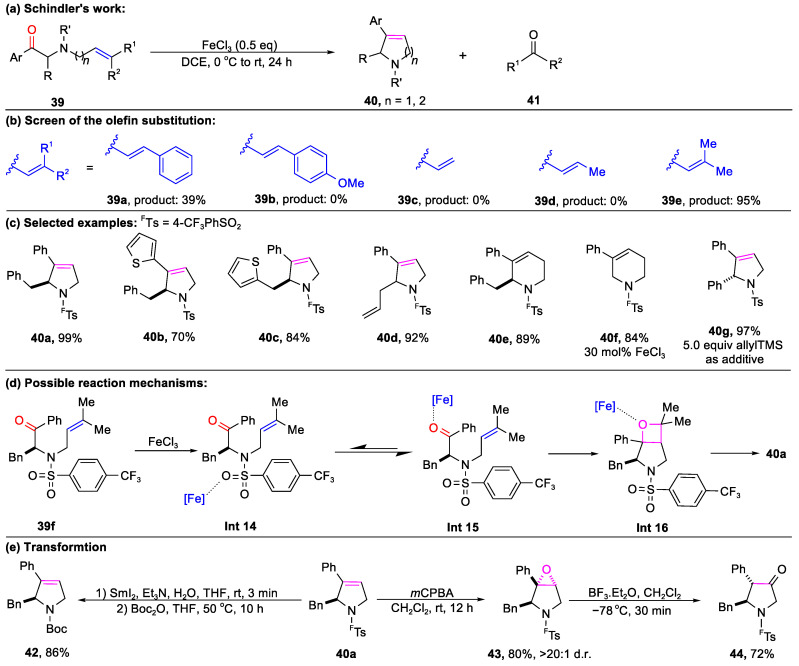
The synthesis of 3-aryl-2,5-dihydropyrroles.

Inspired by previous work, Schindler’s group devised a synthetic route to 3-aryl-2,5-dihydropyrroles via the carbonyl–olefin metathesis of secondary amine **39** (Scheme 7) [58]. This successful transformation was facilitated by the use of 0.5 eq FeCl_3_, which inhibited an efficient turnover and attenuated the Lewis basicity of the sulfonamide moiety, thereby overcoming substrate-induced FeCl_3_ inhibition. However, the unique reaction pathway and stepwise formation of a carbocation intermediate resulted in low yields of the desired product from prenyl-derived alkene substrate **39** [59]. Notably, the expected product could not be observed using **39** with crotyl alkene or terminal alkene as the starting material. Nevertheless, under optimized conditions, a diverse range of 3-pyrroline derivatives incorporating various substituents (**40a**–**40g**) were successfully constructed. Notably, the synthesis of **40g** required the aid of allyl trimethyl silane (5.0 eq). Additionally, substrate **39** containing a methylene substituent in the *α*-position facilitated the formation of the oxetane intermediate. Based on these findings, the authors proposed that the oxygen atom of the sulfonamide and carbonyl group competitively bind to ferric chloride, generating intermediates **Int 14** and **Int 15**. Then, **Int 15** served as a key precursor for promoting the formation of oxetane **Int 16**. Subsequent theoretical investigations demonstrated that an electron-deficient sulfonamide moiety would be beneficial for enabling the turnover of FeCl_3_. Furthermore, under mild conditions, the *t*-butyloxy carbonyl (Boc)-protected derivative of compound **42**, along with pyrrolidine-3-one **44**, could be efficiently constructed.

**Scheme 8:**
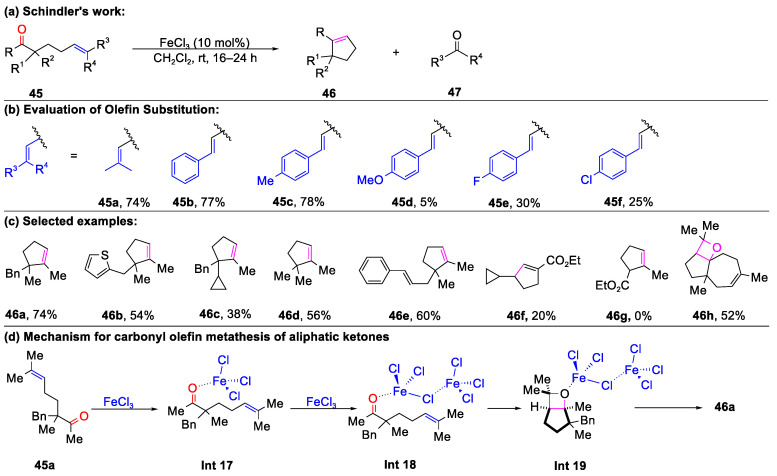
Catalytic carbonyl–olefin metathesis of aliphatic ketones.

In 2019, the same group expanded their work by disclosing a Lewis acidic super-electrophile-mediated carbonyl–olefin metathesis of aliphatic ketone **45** (Scheme 8) [60]. Specifically, allyltrimethylsilane was the most promising additive for restricting this process as a competing product inhibition of the aldehyde by-product was formed, which enabled the carbonyl–olefin metathesis of aliphatic ketones bearing styrene. Diverse substrates, including electron-rich and -deficient aryl, thiophene, cyclopropyl, alkene, and *β*-aliphatic moieties, were tolerated, giving the expected products **46a**–**46f**. However, *β*-ketoester containing aliphatic ketone showed low reactivity (**46f**–**46g**). Interestingly, the oxetane compound **46h** could be isolated in 52% yield. Combining DFT calculations, kinetic studies, Raman spectroscopy, and *IR* data, the researcher proposed a mechanism involving FeCl_3_ coordination, Lewis acid super-electrophile generation, substrate polarization, and fragmentation to form cyclopentene **46a**. Firstly, **Int 17** was afforded by the coordinate of FeCl_3_ with **45a**. Subsequently, a second equivalent of FeCl_3_ was used for the generation of stronger Lewis acid which functioned as a super-electrophile capable of lowering the energy of the transition state to provide sufficient activation for carbonyl–olefin metathesis. Ultimately, the fragmentation of **Int 19** formed by substrate polarization resulted in cyclopentene **46a**.

**Scheme 9:**
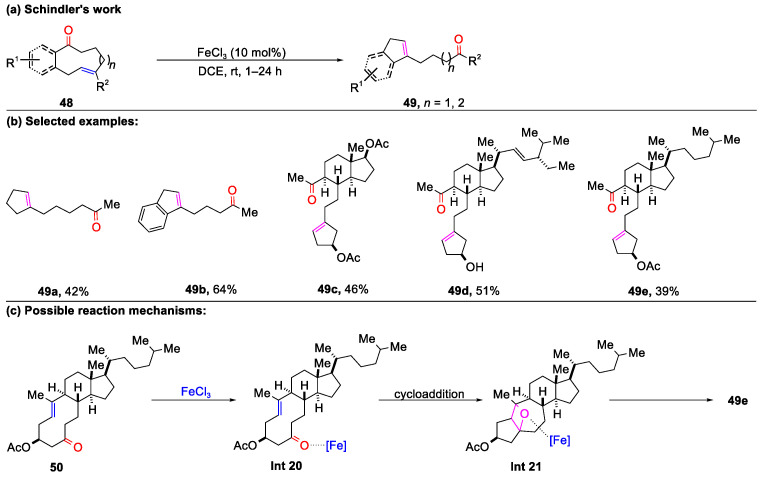
Catalytic, transannular carbonyl–olefin metathesis reactions.

In the same year, it was reported that polycyclic substrate **48** underwent a transan-nular carbonyl–olefin metathesis process, catalyzed by FeCl_3_, to afford multi-substituted cyclopentadiene derivative **49** (Scheme 9) [29]. This conversion was effective for a variety of 9- and 10-membered ring systems, affording the desired metathesis products **49a**–**49e** in moderate yields. Notably, both carbonyl-ene and carbonyl–olefin metathesis pathways competed under FeCl_3_ catalysis, with metathesis prevailing as the thermodynamic product. Importantly, this transformation revealed that either a transannular carbonyl-ene product or carbonyl–olefin metathesis product could be furnished, utilizing a distinct Lewis acid catalyst [61]. Further mechanistic studies indicated that the *retro*-[2+2]-cycloaddition of **Int 21**, formed via a unique reaction pathway involving **Int 20**, offered the expected transannular carbonyl–olefin metathesis product **49e**.

**Scheme 10:**
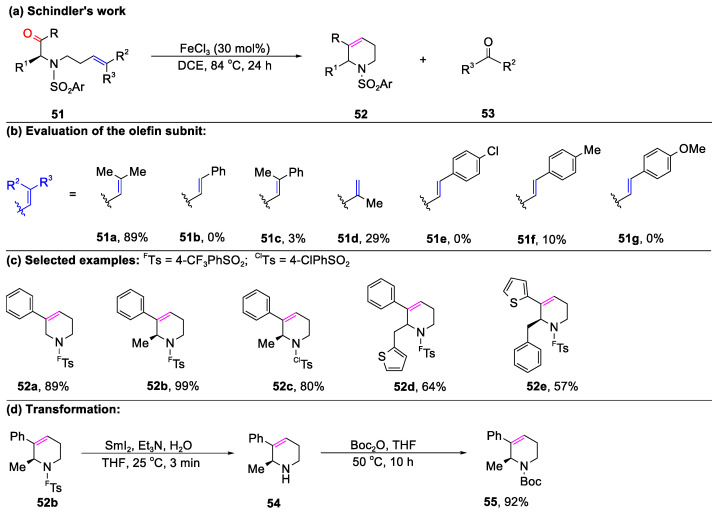
Tetrahydropyridines via FeCl_3_-catalyzed carbonyl–olefin metathesis.

In 2020, a FeCl_3_-mediated carbonyl–olefin metathesis utilizing amino acid **51** as a chiral pool reagent was employed for the synthesis of substituted tetrahydropyridine **52**, accompanied by the formation of by-product ketone **53** (Scheme 10) [62]. Enhanced yields of **52** were realized when using electron-deficient sulfonamide-bearing amino acids **52** as substrates (**52b**, **52c**). The experiments demonstrated that aryl ketone **51** with a prenyl sub-stituent (**51a**) performed optimally, and amino acids featuring alanine, thienyl alanine, and thienyl ketone were well tolerated. Notably, the challenging target compound **52a** was successfully obtained from unsubstituted glycine-derived aryl ketone **51**. Further-more, 1,2,3,6-tetrahydropyridine **55** was accessible from **52b** through deprotection and exposure to Boc_2_O at 50 °C.

**Scheme 11:**
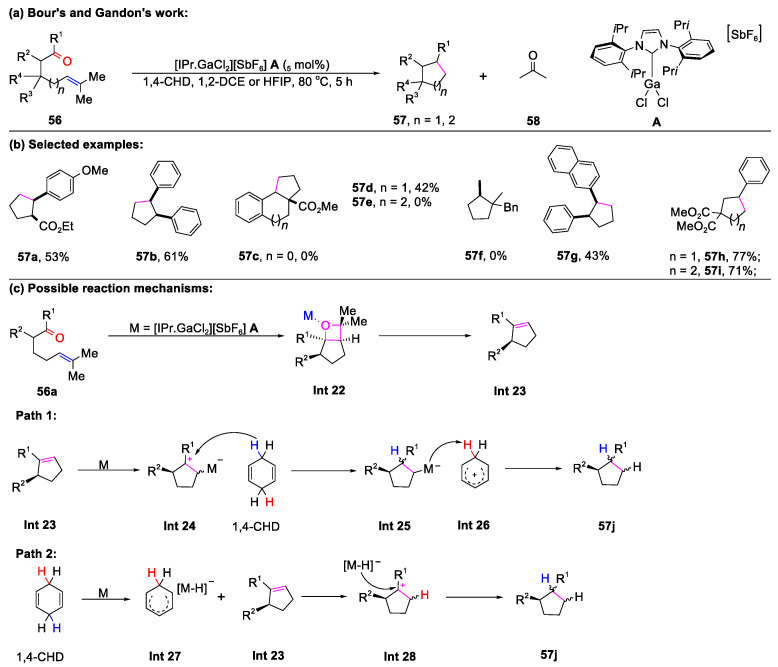
Gallium-catalyzed tandem carbonyl–olefin metathesis/transfer hydrogenation.

In 2019, Bour, Gandon, and their teams developed a series of tandem carbonyl–olefin metathesis and transfer hydrogenation protocols, catalyzed by [IPr·GaCl_2_] [SbF_6_] and uti-lizing 1,4-cyclohexadiene (1,4-CHD) as a H_2_ surrogate, for the synthesis of 1,2-*cis*-disubstituted cyclopentanes and various cyclohexanes (Scheme 11) [63]. This method allowed for the production of various cyclopentanes (**57a**, **57b**, **57g**, and **57h**), tricyclic compound **57d**, and cyclohexane **57i**. Notably, a key advancement was the ability to transform ketone **56** devoid of *β*-substituents into the desired products, contrasting with previous work [54]. While tricyclic **57e** was not observed under optimized conditions, the metathesis product of **57e** was isolated using 1,2-dichloroethane (1,2-DCE) as a solvent. Aliphatic ketones failed to participate in this transformation (**57f**). Building on prior research [54,64,65], it was proposed that the metathesis product **Int 23** originated from [2+2] cycloadduct **Int 22**, proceeding through two distinct pathways to yield **57j**, either via the hydride transfer of carbocation **Int 24** and *proto*-degallation of **Int 25** or through the activation of **Int 28** by a homodimeric Ga-complex **Int 27** coordinated by [IPr·GaCl_2_][SbF_6_] and 1,4-CHD. Finally, the DFT calculations demonstrated path 2 was an advantageous pathway.

**Scheme 12:**
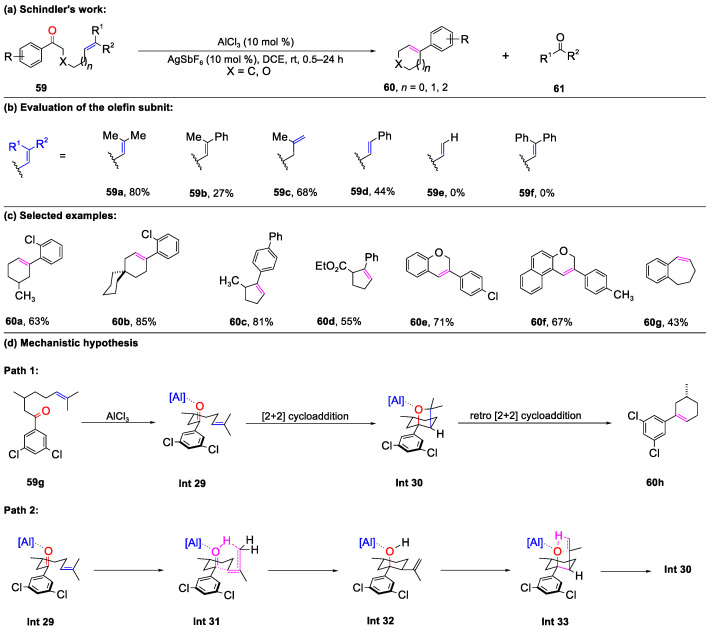
Super-electrophilic aluminum (III)-ion pair-catalyzed carbonyl–olefin metathesis.

Shortly thereafter, Schindler and co-workers disclosed a highly efficient methodology for constructing five-, six-, and seven-membered rings via an aluminum (III)-ion pair-promoted carbonyl–olefin ring-closing metathesis reaction (Scheme 12) [66]. In-depth investigations revealed several key findings: (1) the heterobimetallic ion pairs [AlCl_2_]^+^[SbF_6_]^–^, formed by AlCl_3_ and AgSbF_6_, served as the critical active catalytic species; (2) the unexpected such as benzaldehyde and acetophenone hindered the reaction’s progress; (3) aryl ketones **(59**) containing an oxygen atom in the carbon chain or a *β*-hydrogen atom were well tolerated (**60b**, **60e**, and **60f**); (4) a previously inaccessible seven-membered ring (**60g**) was prepared by this method; and (5) no products stemming from trapped carbocation intermediates were detected through isolation or high-resolution mass spectrometry. Finally, DFT calculations suggested two plausible pathways for the formation of **60h**, involving either a Lewis acid–base complex-mediated cyclization followed by a retro-[2+2] cycloaddition or an intramolecular addition of a terminal alkene via an oxetane intermediate.

**Scheme 13:**
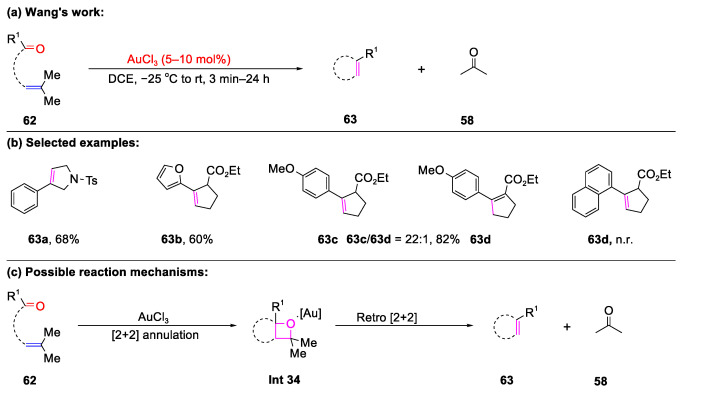
AuCl_3_-catalyzed ring-closing carbonyl–olefin metathesis.

In the same year, Wang’s group realized the AuCl_3_-catalyzed intramolecular ring-closing carbonyl–olefin metathesis of **62**, featuring a pendant isoprenyl group, to produce *N*-heterocycle (**63a**) and cyclopentene (**63b**, **63c**) in good to excellent yields (Scheme 13) [67]. This transformation was compatible with diverse benzene ring backbones containing electron-donating or electron-withdrawing groups, a furan ring, and a *β*-naphthalene ring at R^1^. However, switching *β*-naphthalene with an α-naphthalene ring could not give the corresponding cyclopentene derivative **63d**. Notably, the isomer **63d** is generated using 4-methoxyphenyl-substituted *β*-ketoester as a substrate, which is more selective compared to the approach mediated by FeCl_3_ or Host-HCl [54,68]. Density functional theory (DFT) calculations suggested a [2+2] cycloreversion mechanism of fused oxetane **Int 34,** leading to the desired product **63**.

**Scheme 14:**
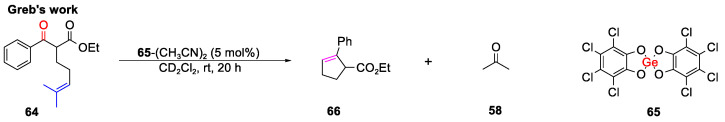
The synthesis of cyclopentene mediated by bis(perchlorocatecholato) germane.

Subsequently, Greb’s group employed *β*-ketoester **64** as a substrate for directly access to cyclopentene **66**, promoted by **65**-(CH_3_CN)_2_ at room temperature in a highly efficient manner, which indicated that the formation of an acetone by-product did not hinder catalytic activity (Scheme 14) [69]. Remarkably, this transformation could not proceed with replacing **65**-(CH_3_CN)_2_ with **65**-(acetone)_2_, which emphasized the critical role of the displacement of the first Lewis base in a *bis*-adduct of **65**.

**Scheme 15:**
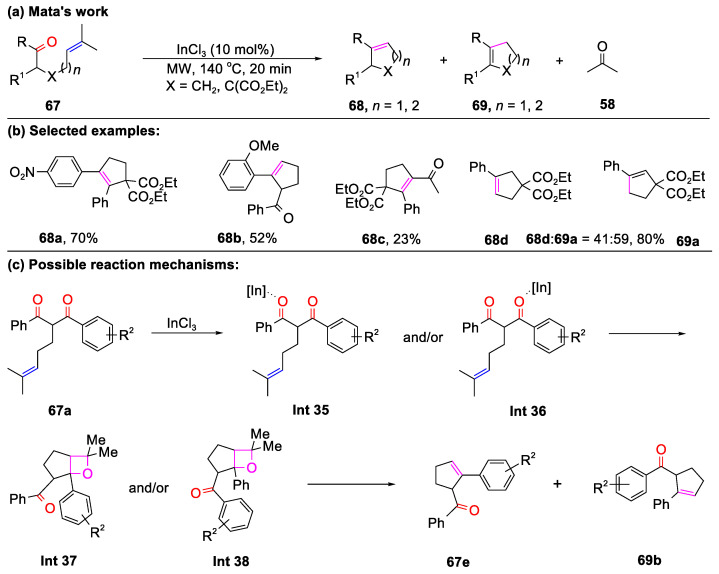
InCl_3_-catalyzed intramolecular carbonyl–olefin metathesis.

In 2023, structurally diverse cyclopentenes **68** and **69** were accessed via ring-closing carbonyl–olefin metathesis, catalyzed by 10 mol% InCl_3_ with the assistance of microwave irradiation (Scheme 15) [70]. Generally, **67** featuring a 1,3-diketone functional group delivered the preferential cyclization of electron-rich aromatic ketones (**68a**–**68b**). While the acetyl and benzoyl groups were contained in the substrate, **68c** was formed in a 23% yield. Mechanistic studies revealed that the tandem [2+2] cycloaddition/cycloreversion process facilitated the construction of **68** and **69**. Notably, the coordination of **67a** with InCl_3_ could occur at the carbonyl group, leading to intermediates **Int 35** and/or **Int 36,** simultaneously forming oxetanes **Int 37** and **Int 38**. Consequently, this reaction provided a mixture of **67e** and **69b**.

### 2.2. Carbonyl–Olefin Metathesis Cyclization Reaction Catalyzed by Metal-Free Condition

**Scheme 16:**
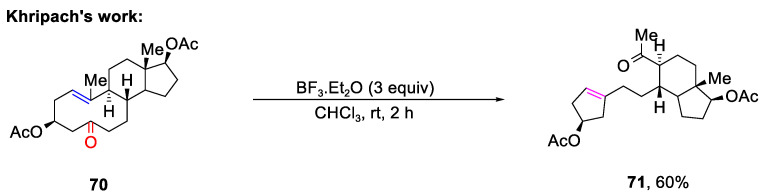
Intramolecular cycloreversion mediated by BF_3_.Et_2_O.

The field of metal-free carbonyl–olefin metathesis emerged as a pivotal branch of research [43,71]. In 2006, the treatment of (*E*)-3*β*,17*β*-diacetoxy-5,10-secoandrost-1(*10*)-en-5-one **70** with BF_3_·Et_2_O was demonstrated by Khripach’s research team, resulting in the cleavage of the macrocyclic structure and the subsequent formation of a novel compound featuring cyclopentenone ring **71** (Scheme 16) [72]. Based on extensive DFT calculations, a plausible reaction mechanism was proposed, involving an intramolecular Lewis acid-promoted [2+2] cycloaddition of a carbonyl with olefin, which was followed by the cycloreversion of the intermediate oxetane species.

**Scheme 17:**
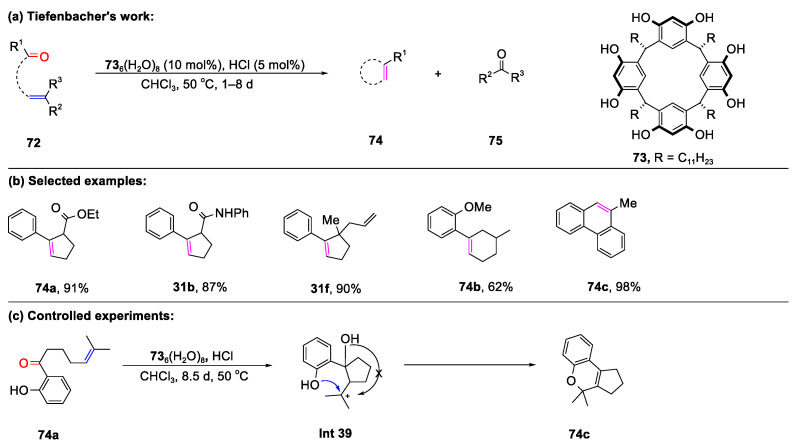
Brønsted acid-catalyzed carbonyl–olefin metathesis.

Subsequently, Tiefenbacher and his colleagues pioneered the first instance of Brønsted acid-mediated carbonyl–olefin metathesis within a self-assembled supramolecular host (Scheme 17) [68]. Their study featured **72** adorned with *α*-methyl styryl, *β*-ketoamide, an unsaturated ketone, a rigid skeleton, and *bis*-substitution at the *α*-position, which proved adept at accessing structurally intricate unsaturated carbon ring frameworks **74a**–**74c**, **31b**, and **31f** in the presence of a co-catalytic system comprising hexamer **73**_6_(H_2_O)_8_ and HCl. It is worth noting that the expected product **74** could not be formed without either **73**_6_(H_2_O)_8_ or HCl. This catalytic system has demonstrated yields that are comparable to, or even surpass, those achieved by the current benchmark Lewis acid. Moreover, the control experiments conducted conclusively demonstrated that the supramolecular host and Brønsted acid collaborate synergistically to promote the metathesis reaction, with the reaction taking place within the confines of the host structure’s cavity [73,74,75]. Under the standard reaction conditions, when probe **74a** was subjected to the process, heterocycle **74c** was exclusively isolated as the sole product of the reaction, providing strong evidence for a stepwise oxetane formation. Compared with previous work [59], it appears likely that the mechanism of the oxetane formation depends on the employed substrate type.

**Scheme 18:**
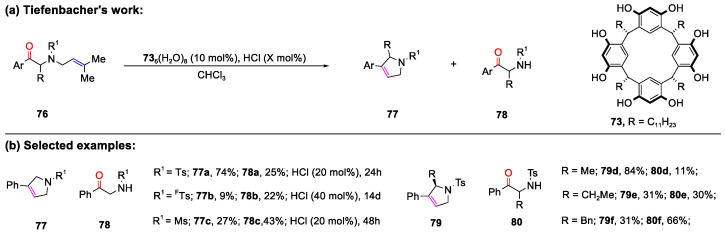
The synthesis of 2,5-dihydropyrroles via carbonyl–olefin metathesis reactions.

In 2022, the same group successfully demonstrated the efficient synthesis of several 3-aryl-2,5-dihydropyrroles (**77**), utilizing the combination of **73**_6_(H_2_O)_8/_HCl (Scheme 18) [76]. This catalytic system was found to be compatible with Lewis basic protecting groups. Additionally, the size of the substrates (**76**) significantly influenced the yield and selectivity of the reaction, which indicated that the carbonyl–olefin metathesis reaction took place inside the capsule exclusively, combined with the result of the control experiments. Intriguingly, the presence of **73**_6_(H_2_O)_8/_HCl triggered the deallylation of chiral amino acid derivatives.

**Scheme 19:**
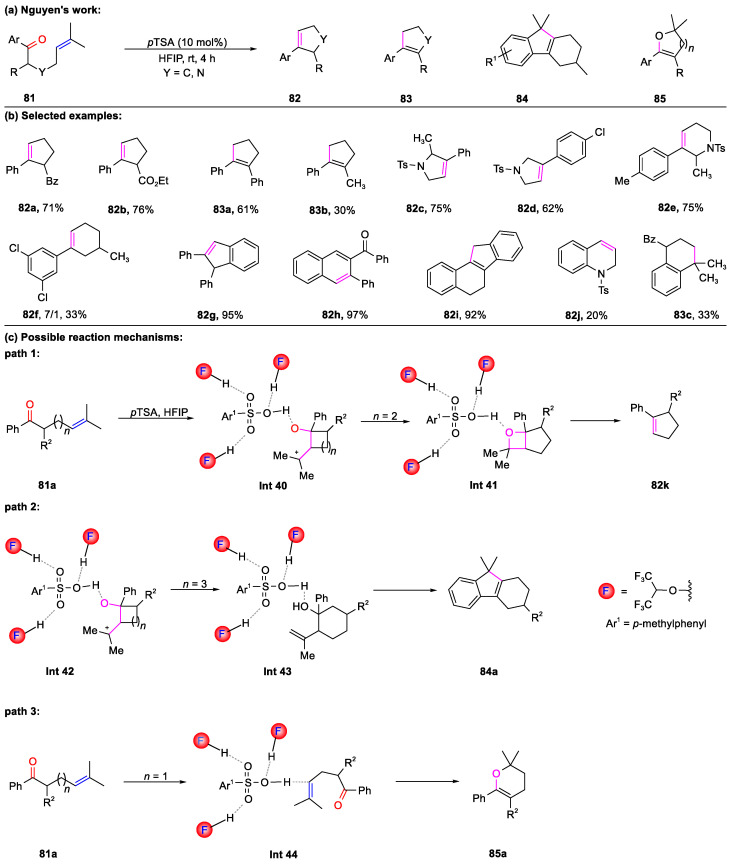
Carbonyl–olefin metathesis catalyzed by HFIP and *p*TSA.

Inspired by these works, Nguyen, Koenigs, and colleagues demonstrated a hydrogen bonding-assisted Brønsted acid catalysis strategy using hexafluoroisopropanol (HFIP) solvent in conjunction with *para*-toluenesulfonic acid (*p*-TSA), which controlled the spatial arrangement of reactants and stabilized the transition state, enabling carbonyl–olefin metathesis reactions to yield a mixture of compound **82** and its isomerized alkene **83** (Scheme 19) [77]. This approach prepared a diverse range of cyclopentenes (**82a** and **82b**), five-membered *N*-heterocycles (**82c** and **82d**), indene (**82g**), naphthalene (**82h**), and six-membered cyclic derivatives (**82e**, **82f**, and **82j**), with good to excellent yields. Moreover, the study demonstrated the feasibility of 6-*endo*-*trig*, carbonyl-ene, and 5-*exo*-*trig* cyclization processes, affording desired products **84a** and **85a** in moderate yields. The theoretical analysis revealed the following: (1) HFIP played an important role in the formation of hydrogen bond networks; (2) the proton migration process resulted in **Int 43,** which delivered classic carbonyl-ene product **84a** via a stepwise mechanism; (3) when n = 1 in **81a**, the high ring strain of the putative 1-*oxo*-bicyclo-[*2.2.0*]-hexane intermediate could not be formed smoothly, while the 6-*endo dig* cyclization reaction giving pyrane **85a** was a good choice; and (4) **Int 44** without an activated carbonyl group underwent 6-*endo*-*trig* and 5-*exo*-*trig* cyclization, leading to the formation of **85a**. While there was an aromatic substituent at the *alpha* position of **81a**, cyclization in a Friedel–Crafts alkylation fashion worked smoothly, affording tetrahydronaphthalene **83c**.

**Scheme 20:**
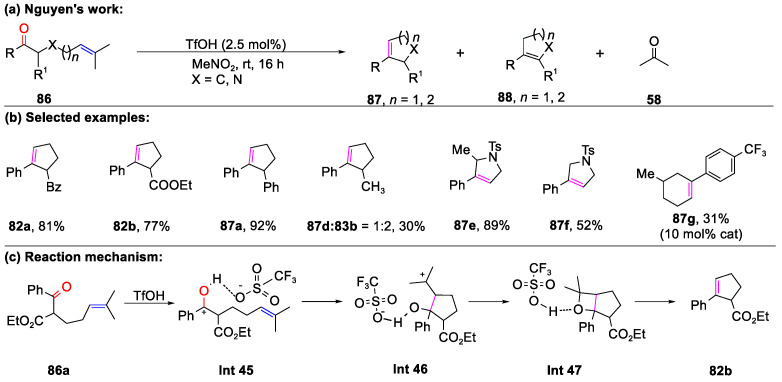
Brønsted acid-catalyzed intramolecular carbonyl–olefin metathesis reactions.

Very recently, a homogeneous Brønsted acid-catalyzed intramolecular ring-closing protocol employing nitromethane as a solvent was established by the same group (Scheme 20) [78]. It is worth noting that a negligible isomerization product was observed in this transformation. Diverse cyclopentenes (**82a**, **82b**, **87a**, and **87d**) and five-membered heterocycle derivatives (**87e** and **87f**) were successfully constructed. Compared with **82a** and **87d**, the substituent at the *α*-position of **86** has a significant impact on the yield of the isomerization. Further investigations revealed that protonated carbonyl species (**Int 45**) derived from triflic acid transformation underwent a stepwise mechanism involving two nucleophilic addition steps, leading to the formation of the oxetane intermediate **Int 47** via an ion pair intermediate **Int 46**. This was followed by a retro-[2+2]-cycloaddition of **Int 47**, resulting in the cycloalkene product **82b**.

**Scheme 21:**
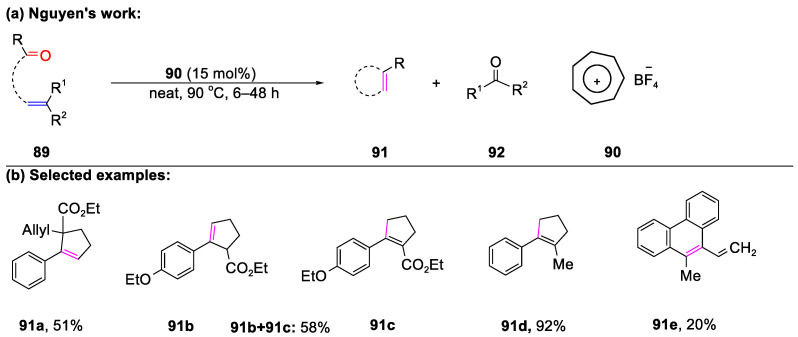
Tropylium-promoted carbonyl–olefin metathesis reactions.

In 2018, Nguyen introduced a novel approach leveraging tropylium ions (**90**) to foster carbon–carbon bond formation, enabling both intramolecular and intermolecular carbonyl–olefin metathesis, as well as ring-opening metathesis (Scheme 21) [79]. This method accommodated a diverse array of substrates, giving the anticipated products (**91**), albeit with the inevitable isomerization of newly formed double bonds (**91b** and **91c**). Notably, **89** featuring methyl substituents at the *α* position relative to the original carbonyl group typically undergoes a rearrangement to offer thermodynamically stable olefin product **91d**. These results of the control experiments suggested that the volatility of acetone may play a role in driving the reaction equilibrium towards the formation of **91**. Mechanistically, the Lewis acidity of tropylium **90** was instrumental in catalyzing these metathesis reactions.

**Scheme 22:**
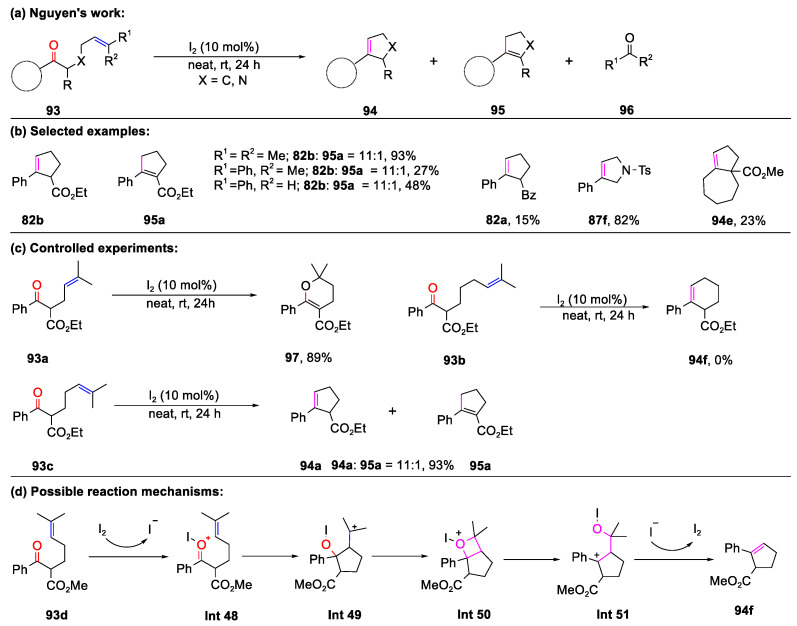
Carbonyl–olefin metathesis catalyzed by molecular iodine.

Later, carbonyl–olefin metathesis promoted by molecular iodine was unveiled by Nguyen and co-workers (Scheme 22) [80]. When non-nitrogen-containing substrate **93** underwent this transformation, a mixture of regioisomeric products (**94** and **95**) was observed due to trace Brønsted acids in the reaction mixture. Notably, the functional group at the α-position of the new C–C double bond have an influence on the ratio of **94** to **95**. For example, for substrates containing Brønsted basic nitrogen centers or double substitution at the *α*-position of **93**, isomerization would not occur (**87f**, **94e**). Moreover, the conjugation between the aromatic ring and the carbonyl group is crucial for this transformation, which could explain the low yield of **94e**. Increasing the loading to 25%, the corresponding product **94c** could be obtained through the metathesis of **93,** without using a substitution on the carbon between the carbonyl and the nitrogen centers as a substrate. With different carbon chain linkers between the carbonyl and the olefin moieties, the target compound changed vastly (Scheme 22c). Based on these controlled experiments, DFT calculations and kinetic studies, Nguyen’s group hypothesized that I^+^ ion produced by a very endergonic splitting of molecular iodine due to an unfavorable charge separation could have served as a Lewis acid to activate the carbonyl group providing **Int 48**, which triggered the formation of oxetane **Int 50**, followed by a rearrangement, generating **94f**.

**Scheme 23:**
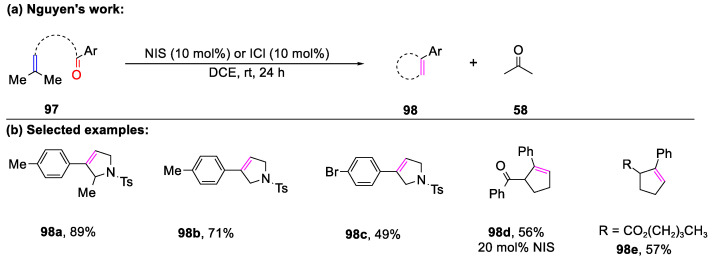
Iodonium-catalyzed carbonyl–olefin metathesis reactions.

At the same time, a similar experiment where *N*-iodosuccimide (NIS) and iodine monochloride (ICl) were engaged as iodonium sources to promote the intramolecular carbonyl–olefin metathesis of **97** was achieved (Scheme 23) [81], further expanding the toolbox for these transformative reactions. Interestingly, the noticeable regioisomerization of the cyclized product **98** was not observed in this conversion. The control experiment validated that the iodonium catalytic pathway of molecular iodine promoted this process.

## 3. The Formation of Bicyclic Compounds

### 3.1. The Formation of Bicyclic Compounds Catalyzed by Metal

**Scheme 24:**
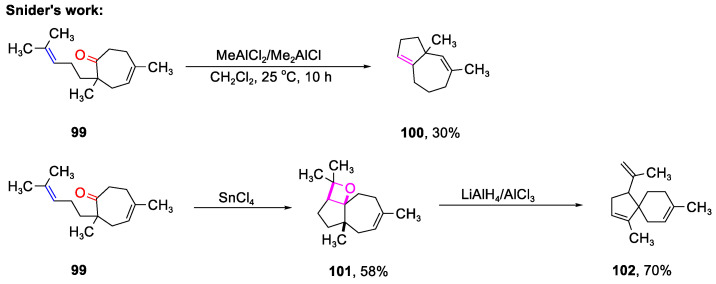
Al-catalyzed carbonyl–olefin metathesis reactions.

In 1984, a 30% yield of 7,8-dimethyl-1,2,4,5,6,8-hexahydroazulene **100** was achieved through the ring-closing process of **99**, involving the loss of acetone, in the presence of a specific ratio of MeAlCl_2_ (2/3 eq) and Me_2_AlCl (1/3 eq) (Scheme 24) [82], which opened the door to the synthesis of bicyclic structures through a Lewis acid-catalyzed carbonyl–olefin metathesis reaction. Notably, when **99** was treated with SnCl_4_, LiAlH_4_, and AlCl_3_, a spirocyclic product, **102,** was obtained instead [83], highlighting the significant influence of the chosen Lewis acid on product formation.

**Scheme 25:**
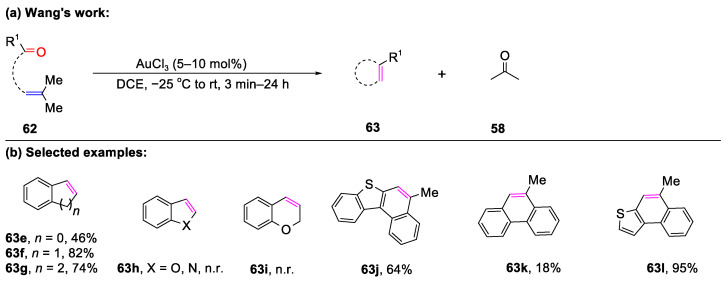
Au-catalyzed carbonyl–olefin metathesis reactions.

Inspired by Snider’s work, various bicyclic frameworks were provided, mediated by FeCl_3_ and AlCl_3_ (**56**, **57**, and **69**). In 2020, Wang and co-workers described an efficient way to construct bicyclic aromatic skeletons catalyzed by AuCl_3_ (Scheme 25) [67]. Moreover, 1*H*-indene **63e**, 1,2-dihydronaphthalene **63f**, and 6,7-dihydro-5*H*-benzo [7] annulene **63g** were prepared through this process. Unfortunately, indole **63h**, benzofuran **63h**, and 2*H*-chromene **63i** could not be obtained through carbon–oxygen and carbon–nitrogen bond cleavages promoted by AuCl_3_. Importantly, polycycle derivatives (**63j**–**63l**) were delivered in good yields.

**Scheme 26:**
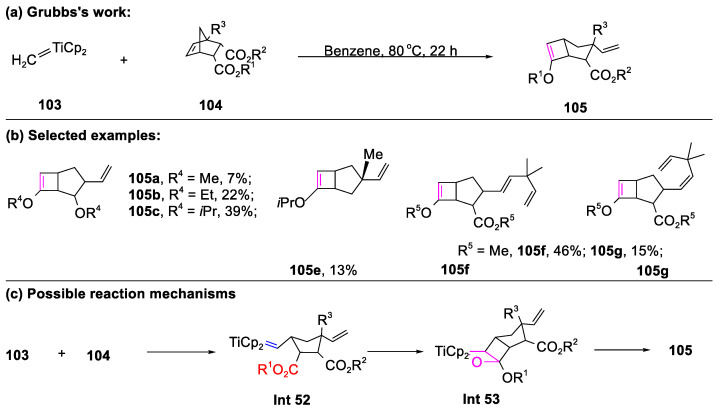
Titanocene alkylidene complex-catalyzed carbonyl–olefin metathesis reactions.

**Scheme 27:**
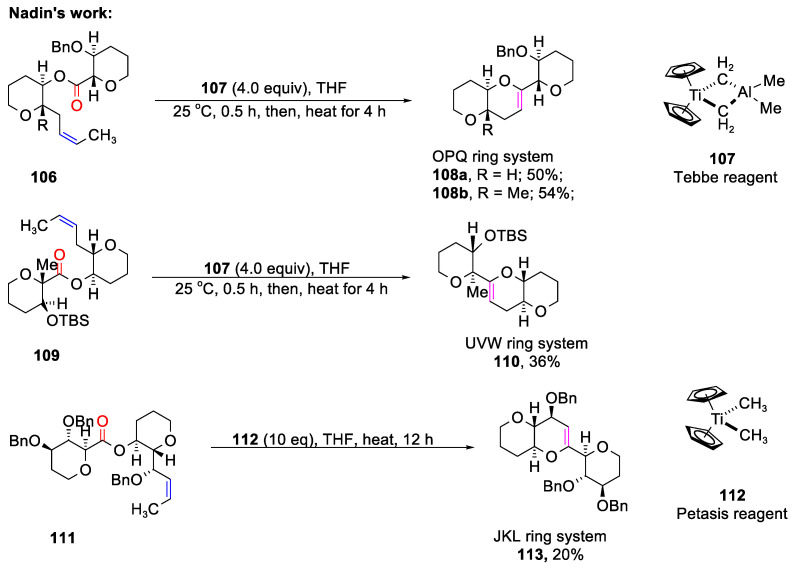
The construction of the JKL, OPQ, and UVW ring systems of Maitotoxin.

Moreover, it was realized that ester-substituted norbornenes (**104**), in conjunction with Titanamethylene complex **103**, served as substrates to synthesize bicyclo[3.2.0]heptene ring **105** via cyclobutane intermediate **Int 52** (Scheme 26) [46]. Enhancing the steric bulk of the ester group not only fortified steric protection around the carbonyl, limiting methylene transfer, but also intensified vicinal steric interactions between the ester groups, promoting the norbornene metathesis process (**105a**–**105c**). Additionally, the carbonyl–olefin metathesis of unsymmetrical norbornene substrates facilitated the production of desired products (**105e**–**105g**) in moderate yields, without a competing reaction between the norbornene double bond and *endo*-ester. Moreover, the construction of OPQ, UVW, and JKL ring systems found in maitotoxin was achieved through the carbonyl–olefin metathesis reactions of **106, 109,** and **111**, followed by a reduction in the resulting carbon–carbon double bonds catalyzed by Tebbe reagent **107** or Petasis reagent **112** (Scheme 27) [84].

**Scheme 28:**
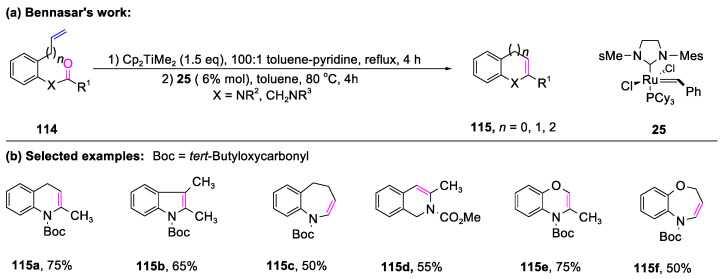
Approach to 1,4-dihydroquinoline via carbonyl–olefin metathesis reactions.

Bennasar’s group explored an innovative two-step procedure that involved the titanium-mediated methylation of *N*-acylamides (**114**), followed by ruthenium-catalyzed ring-closing metathesis (RCM) to give enamide **115** (Scheme 28) [85,86]. This protocol efficiently facilitated the synthesis of various benzo-fused five- and six-membered cyclic enamides (**115a**–**115f**) (including indole, 1,4-dihydroquinoline, and 1,2-dihydroisoquinoline) from an amide derived from *ortho*-alkenyl aniline or benzylamine. While there was potential for an extension to higher homologues, this process was constrained by the interference of alkene isomerization during the sluggish RCM step. Notably, direct annulation was observed in the titanium-mediated step, particularly with certain substrates, such as styrene derivatives, likely occurring through an olefin metathesis–intramolecular olefination tandem mechanism.

**Scheme 29:**
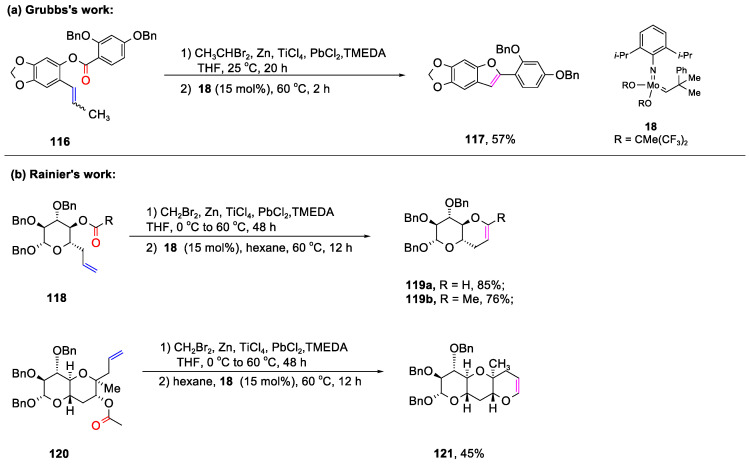
Approach to fused ether ring systems via carbonyl–olefin metathesis reactions.

Drawing inspiration from Clark’s work [87,88], Grubbs and Rainier’s teams devised a method for synthesizing benzofuran derivative **117** and cyclic enol ethers (**119** and **121**) (Scheme 29) [89,90]. This method involved the use of stoichiometric titanium reagents to convert esters into acyclic olefinic enol ethers, which were subsequently transformed into the desired products through catalytic ring-closing olefin metathesis utilizing molybdenum alkylidene complex **18**.

**Scheme 30:**
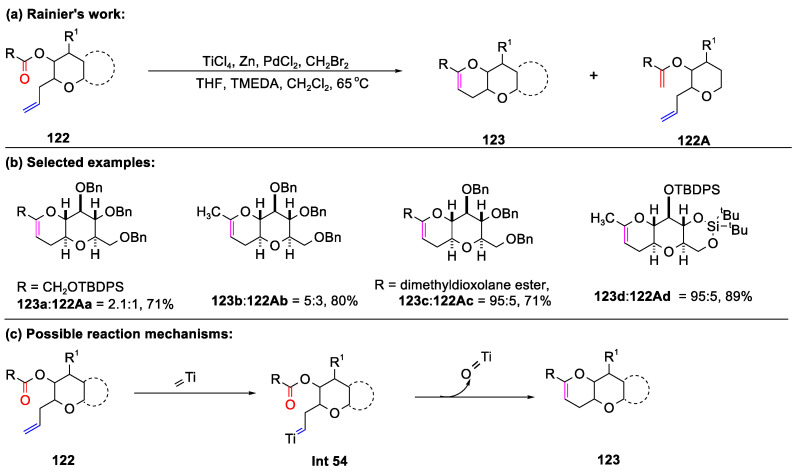
Olefinic ester cyclizations using Takai–Utimoto-reduced titanium alkylidenes.

**Scheme 31:**
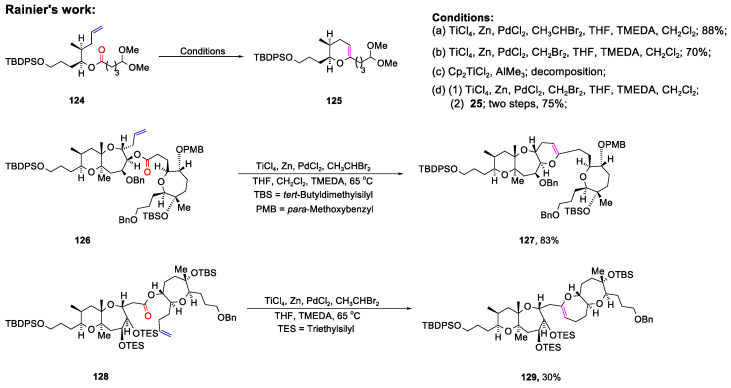
Ti alkylidenes mediated carbonyl–olefin metathesis reactions.

Excitingly, Rainier and colleagues demonstrated that Takai–Utimoto-reduced titanium alkylidene could promote the ring-closing reaction of olefinic esters (**122**), directly producing bicyclic compound **123** in high yields (Scheme 30) [91]. Notably, the steric environment of the ester or olefin influences the ratio of cyclic enol ethers **123** to **124**. Moreover, the olefin metathesis of **122** could proceed under standard conditions. Moreover, this conversion was realized via the Ti intermediate **Int 54** generated in situ. Given the advantages of this method, it was applied for the synthesis of key molecular building blocks (**125**, **127**, and **129**) to construct Brevenal, which demonstrated the practicality of the olefinic ester cyclization reactions (Scheme 31) [39].

**Scheme 32:**
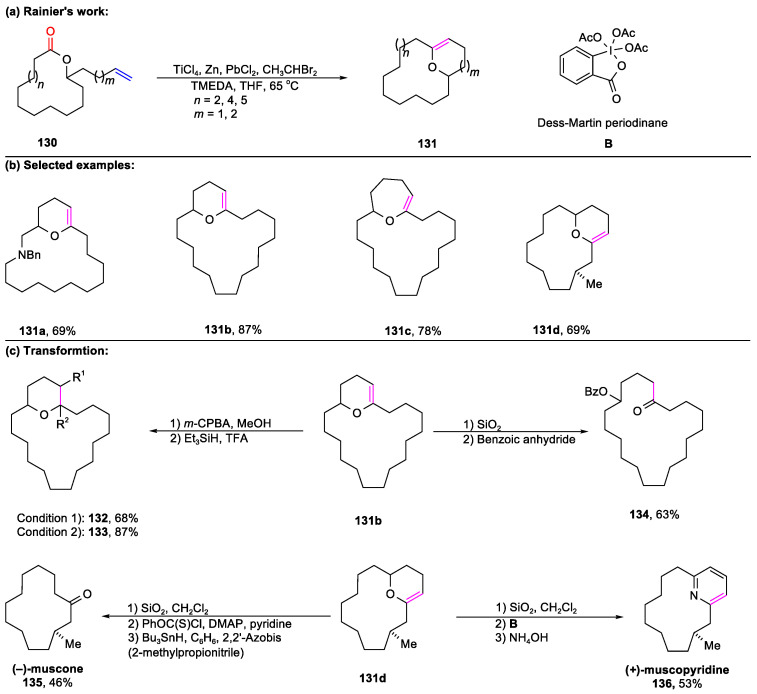
Olefinic lactone cyclization to macrocycles.

As a continuous study, olefinic lactone cyclization to macrocycle **131** under similar conditions was developed in 2009 (Scheme 32) [92]. Employing 13-, 16- or 17-membered lactones as starting materials, the desired products **131a**–**131d** were constructed. In addition, the oxidation, reduction, and ring-opening process of **131** proceeded smoothly to give macrocyclic ketal **132**, macrocyclic pyran **133**, and macrocyclic ketone **134** in good yields. Furthermore, natural products (*R*)-(*−*)-muscone **135** and (*R*)-(+)-muscopyridine **136** could be delivered through the key intermediate **131d**.

**Scheme 33:**
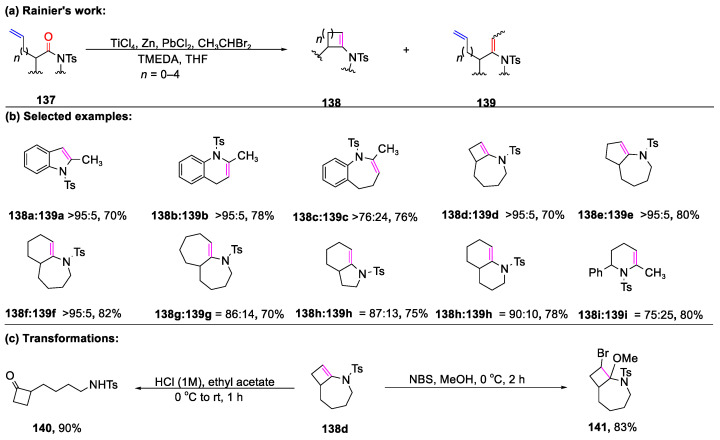
Olefinic amide and olefinic lactam cyclizations.

The use of in situ-generated titanium ethylidene to react with olefinic amide or olefinic lactam, facilitating indole **138a**, dihydroquinoline **138b**, and fused seven-membered ring **138c,** has been reported by the same group (Scheme 33) [93]. The carbonyl–olefina tion pathway of nonaromatic olefinic actam **137** could proceed to provide **138d**–**138i**. Notably, these transformations were accompanied by an olefin metathesis reaction to give **139**, which could be inhibited by using dihydroquinone as an additive [94]. Moreover, the treatment of **138d** with aqueous acid would convert it into cyclobutanone **140**. Bromoaminal **141** was afforded by the olefin functionalization of **138d**. Further experiments illustrating the carbonyl-ene alkylation of **137** could not furnish the desired product, **138,** under optimized reaction conditions.

### 3.2. The Formation of Bicyclic Compounds Catalyzed by Metal-Free Conditions

**Scheme 34:**
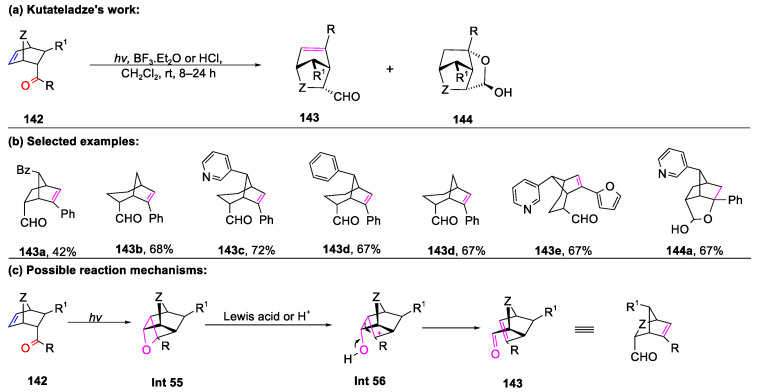
Photoprotolytic-mediated carbonyl–olefin metathesis reactions.

In 2009, Kutateladze’s research team disclosed a highly efficient methodology involving a one-pot, photoinduced transformation of *endo*-aroyl and heteroaroyl Diels–Alder adducts (**142**), facilitating the construction of novel bicyclic aldehydes (**143**) or their corresponding hemiacetals (**144**), with satisfactory yields (Scheme 34) [24]. Bicyclic aldehyde **143** adorned with carbo- and heterocyclic compounds could be obtained through this transformation. The targeted product, **143,** was attained through a sequence comprising the Paternò–Büchi reaction, a subsequent Grob-like fragmentation of the initially ring-opened carbocationic intermediate **Int 56**, and ultimately, acidic conditions promoting the formation of hemiacetal **144** via a secondary electrophilic addition. Notably, trace amounts of HCl present in solvents like dichloromethane could modulate the cycloreversion process. Furthermore, the oxetane intermediate **Int 55** could be diverted towards aldehydes or hemiacetals, contingent upon the utilization of Lewis acid or protic acid.

**Scheme 35:**
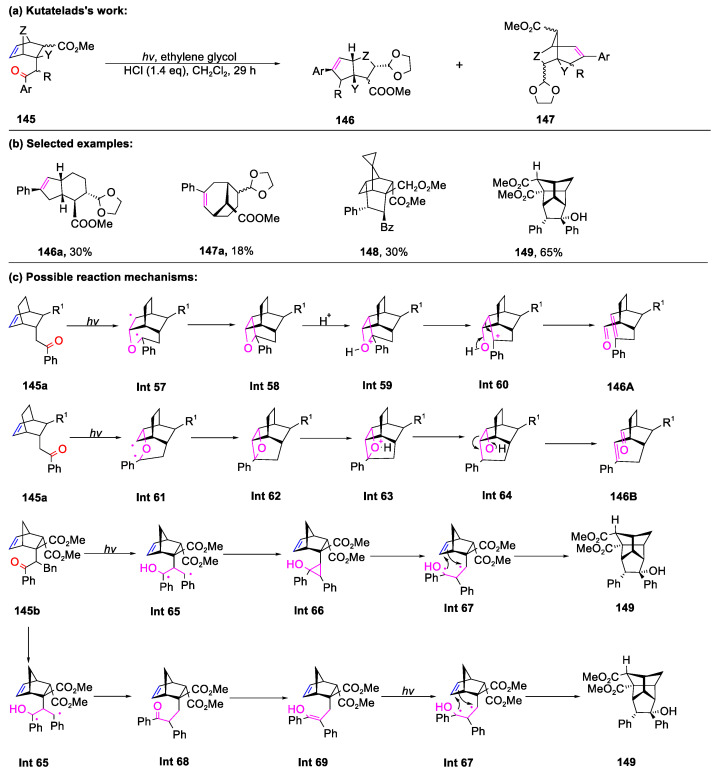
The synthesis of bicyclic compounds via photoinduced intramolecular cyclization.

In 2013, the same group reported that conformationally pliable aroyl methyl chromophore-decorated bicyclic scaffolds (**145**) were harnessed to construct innovative bicyclic frameworks **146** and **147**, leveraging photoinduced intramolecular cyclo-penetration and photo-protolytic oxametathesis reactions (Scheme 35) [95]. Subsequent investigations highlighted several crucial insights as follows: (1) the steric hindrance of **145** significantly influenced the ratio of intermediates **Int 57** to **Int 61**; (2) the epimerization of **146A** and **146B** via enolization, bringing a formyl group into the *exo*-position, was restricted in the presence of ethylene glycol; (3) *β*-hydrogen abstraction from the CH_2_ group by an excited benzoyl moiety proved pivotal for the formation of **149**; (4) this unique photorearrangement/cyclo-penetration paradigm involving double bonds emerged as an effective strategy for synthesizing fused cyclopentanol. Notably, **Int 68** was converted from **Int 65,** involving a 1,2-radical shift of the bicyclic core facilitated by enol formation, followed by the keto-enol equilibrium, second excitation with H abstraction, and an interrupted cyclization to give **149**.

**Scheme 36:**
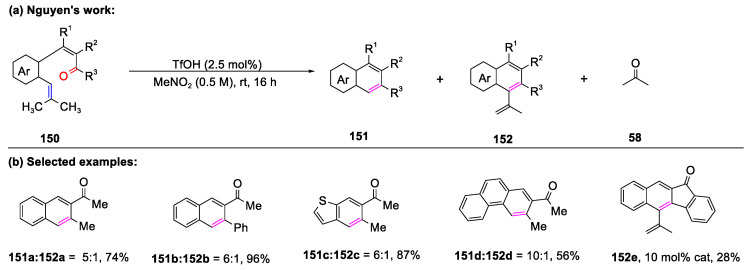
The synthesis of bicyclic compounds mediated by TfOH.

Recently, trifluoromethanesulfonic acid (TfOH)/MeNO_2_ was proved to be an efficient combination to promote the carbonyl–olefin metathesis (COM) reaction of **150**, producing various polyaromatic products (**151a**–**151d** and **152a**–**152e**) (Scheme 36) [78]. Notably, small amounts of carbonyl-ene-type by-products (**152**) were inevitably generated.

**Scheme 37:**
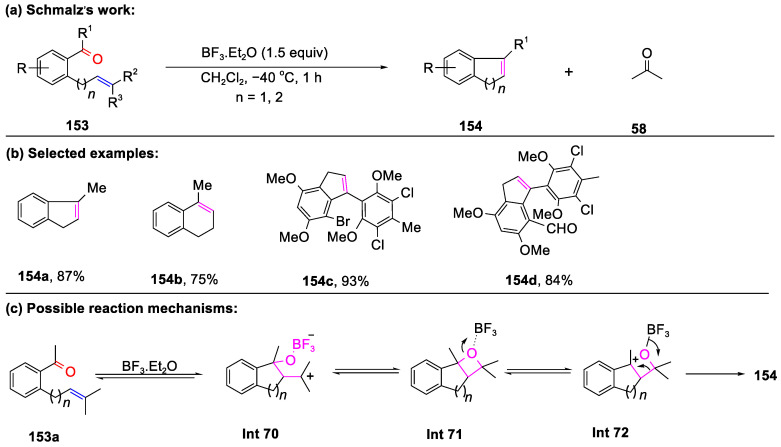
BF_3_.Et_2_O mediated intramolecular carbonyl–olefin metathesis.

Additionally, Schmalz and his collaborators established a protocol involving boron trifluoride etherate-mediated intramolecular carbonyl–olefin metathesis (Scheme 37) [96]. In this process, acetophenone or benzophenone derivative **153** underwent Prins-type (*exo*-*trig*) cyclization, forming a tertiary carbenium ion, **Int 70**, which subsequently isomerized to **Int 72** via oxetane intermediate **Int 71**. Ultimately, the fragmentation of **Int 72** produced the desired product **154**, with yields reaching 93%, accompanied by ketone **58** as a by-product.

**Scheme 38:**
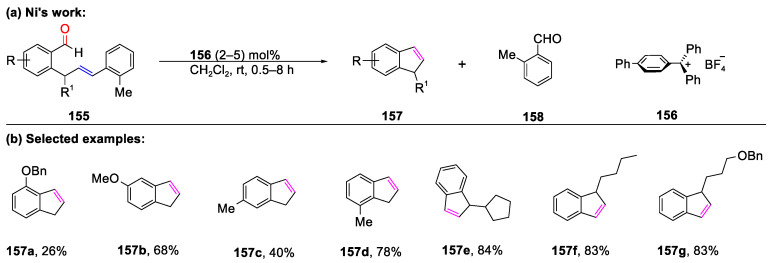
Carbocation-catalyzed ring-closing aldehyde–olefin metathesis.

Subsequently, 4-phenyl-diphenylmethylium ion **156** proved to be an adept catalyst for promoting the ring-closing metathesis of aldehyde and olefin, leading to the synthesis of indene **157** in good to excellent yields (Scheme 38) [97]. The loading of **156** could be reduced to 2 mol%, and these products (**157**) could be isolated in good yields, with high purity and selectivity, which is different from previous work. Utilizing the developed method, a series of functionalized indene derivatives with various substituents at any position could be easily prepared. Importantly, the decomposition of product **157** was observed under optimized reaction conditions, and the benzylic functionalization of enal **155** was compatible with this transformation, facilitated by the addition of just 2 mol% of **156**.

**Scheme 39:**
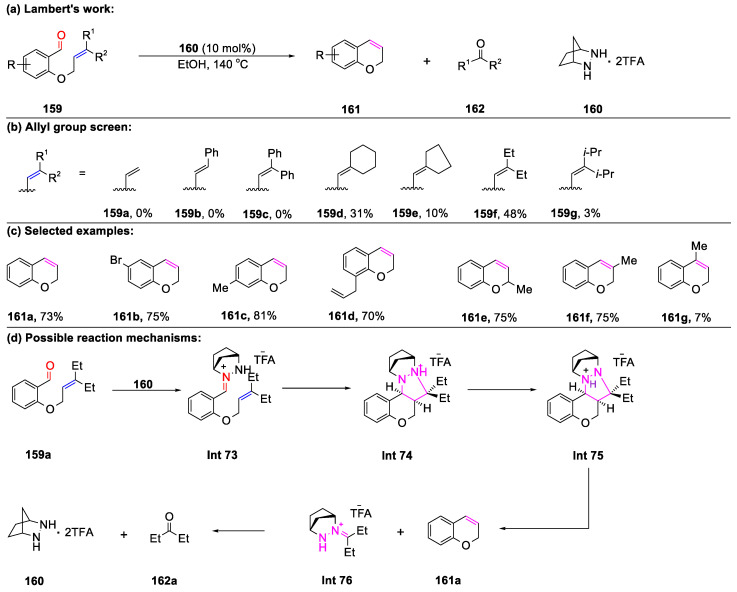
Hydrazine-catalyzed ring-closing carbonyl–olefin metathesis.

Later, the synthesis of 2*H*-chromene **161** was successfully achieved through a novel [3+2]/retro-[3+2] metathesis sequence, utilizing *O*-allyl salicylaldehyde **159** as the substrate, catalyzed by 10 mol% of [2.2.1]-bicyclic hydrazine **160** (Scheme 39) [98]. Among the various allyl groups screened, 3,3-diethylallyl emerged as the optimal fragment for this transformation, effectively suppressing undesired deallylation side reactions. Notably, compound **159** with substitutions at positions 6, 7, and 8 was suitable for this transformation, enhancing the versatility of this process. Increasing the loading of **166** to 20 mol% and replacing ethanol with isopropanol enable the smooth formation of **161e**. Critically, a formal high-throughput intermolecular screening method was implemented to assess the compatibility of various functional groups, demonstrating robust tolerance towards ketones, unprotected alcohols, and carboxylic acids [99]. A plausible reaction mechanism was then proposed, containing the condensation of aldehyde **159a** with hydrazine **160** to form hydrazonium intermediate **Int 73**, which subsequently underwent 1,3-dipolar cycloaddition with an olefin to offer **Int 74**. Following proton migration and cycloreversion, 2*H*-chromenes (**161a**) were furnished, with hydrazine **160** being regenerated through the hydrolysis of **Int 76.** Additional DFT calculations underscored the importance of allyl substitution patterns in sterically hindered systems, where cycloreversion was facilitated by the relief of the *syn*–pentane strain in the diethylidene moiety.

**Scheme 40:**
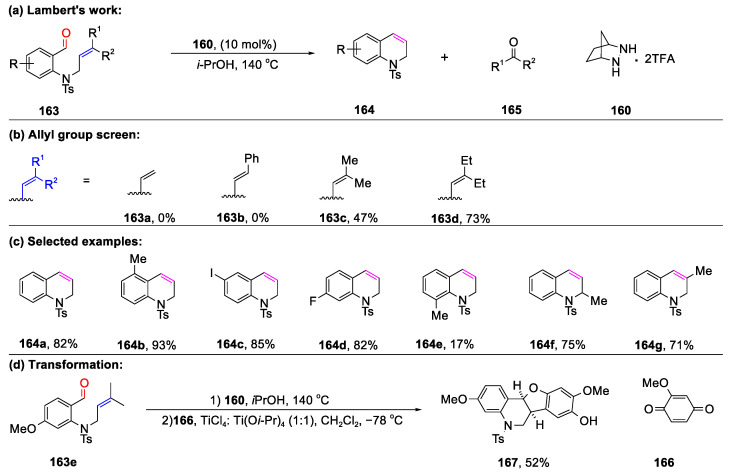
Synthesis of 1,2-dihydroquinolines via ring-closing carbonyl–olefin metathesis.

In 2020, the same group utilized a similar strategy to construct 1,2-dihydroquinoline **164**, utilizing *N*-prenylated 2-aminobenzaldehyde **163** as the substrate (Scheme 40) [100]. While the cycloreversion step was found to proceed more rapidly compared to the *O*-allyl salicylaldehyde system, the formation of the cyclization intermediate was hindered by the strong electron-donating capability of the nitrogen atom. Employing *t*-butyloxycarbonyl (Boc) or acetyl (Ac) as *N*-protecting groups led to the formation of quinoline derivatives. However, a pronounced steric clash between the tosyl group and methyl substituent negatively impacted the yield of **164e**. Additionally, the presence of aniline and alkyl halide additives was detrimental to the product yield, attributed to their competitive reactions with **160** or an aldehyde. Nonetheless, the optimization of reaction conditions followed by Lewis acid-mediated cycloaddition allowed for the isolation of the azapterocarpan analog **167** in a respectable 52% yield [101].

## 4. The Formation of Polycyclic Compounds

### 4.1. The Formation of Polycyclic Compounds Catalyzed by Metal

**Scheme 41:**
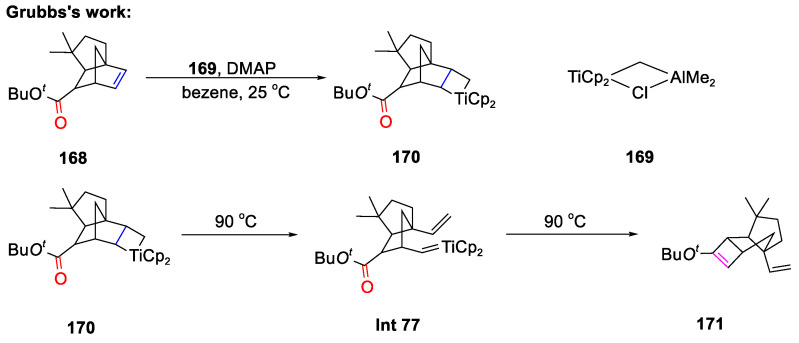
Ring-closing carbonyl–olefin metathesis catalyzed by titanium reagents.

**Scheme 42:**
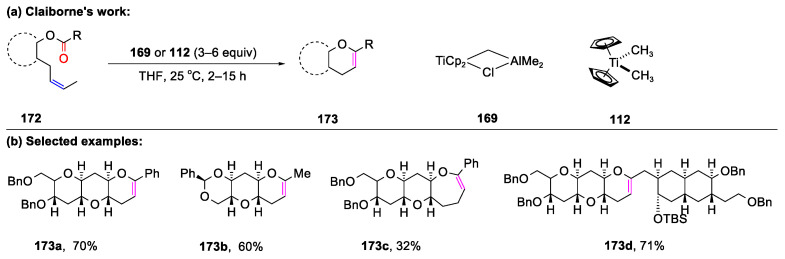
Olefin metathesis in cyclic ether formation.

The synthesis of polycyclic structures has been a formidable challenge in chemistry. In 1986, Grubbs pioneered an effective approach to accessing capnellane, wherein substrate **168** underwent an intricate intramolecular cycloaddition, ring-opening, and olefin metathesis sequence in the presence of titanium reagent **169** and DMAP, providing the polycyclic product **171** (Scheme 41) [102]. Subsequently, capnellane was derived from **171** through a five-step synthesis. Further developments indicated olefin esters could be directly converted into enol ethers featuring polycyclic structures with six- or seven-membered rings in high yields, facilitated by Tebbe (**169**) or Petasis reagents (**112**) (Scheme 42) [45]. The proposed mechanism commenced with the formation of titanacyclobutane, resulting from the olefination of enol ether **172** with **169** or **112**. This intermediate then underwent fragmentation, intramolecular cycloaddition, and regioselective fragmentation, ultimately leading to the desired product **173**. Additionally, hexacyclic polyethers could be synthesized to form **173d** through three steps.

**Scheme 43:**
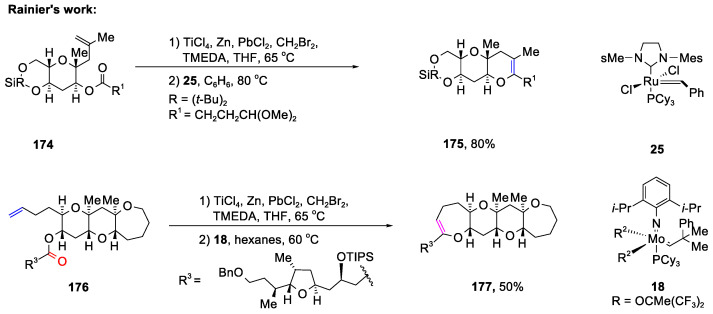
The application of olefin metathesis.

**Scheme 44:**
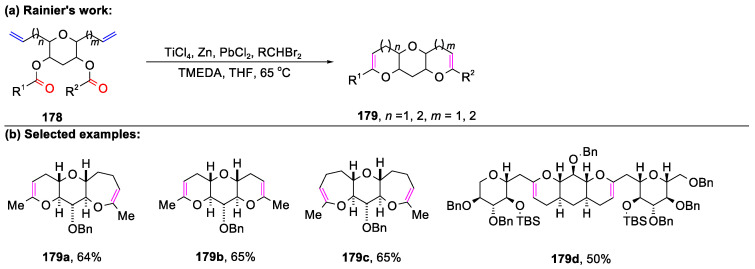
Two-directional olefinic ester ring-closing metathesis.

**Scheme 45:**
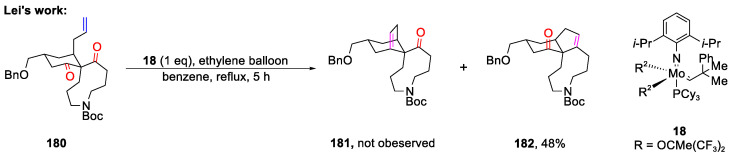
The synthesis of tricycle compound.

Using a Ti reagent, a precursor **174** adorned with a silicon ring could undergo ether–olefin ring-closing metathesis in the presence of **25**, giving the expected product **175** in an 80% yield (Scheme 43) [103]. Moreover, **177,** containing double seven-membered rings, was also produced by this methodology in a 50% yield utilizing Schrock catalyst **18**. In 2009, the same research group disclosed a novel two-directional olefinic ester ring-closing metathesis promoted by reduced Ti alkylidenes (Scheme 44) [104], enabling the synthesis of tri-, penta-, and heptacyclic scaffolds (**179a**–**179d**). When the starting material (**180**) possessed two carbonyl groups, direct metathesis between the alkene and carbonyl moieties yielded 48% of **182**, with the absence of competitive product **181** attributed to the high torsional strain in the bridged structure (Scheme 45) [105]. Moreover, (–) huperzine could be prepared through four steps with **182** as the starting material.

**Scheme 46:**
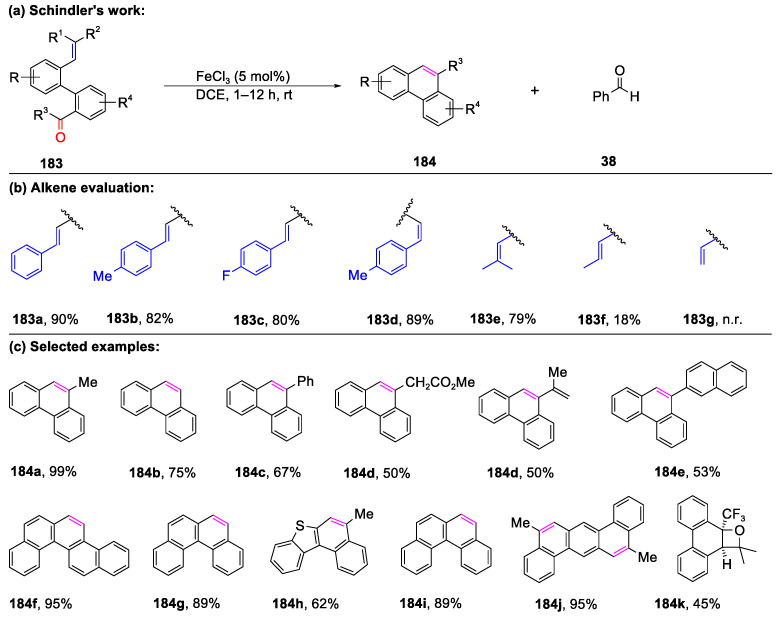
Polycyclic aromatic hydrocarbons via iron(III)-catalyzed carbonyl–olefin metathesis.

**Scheme 47:**
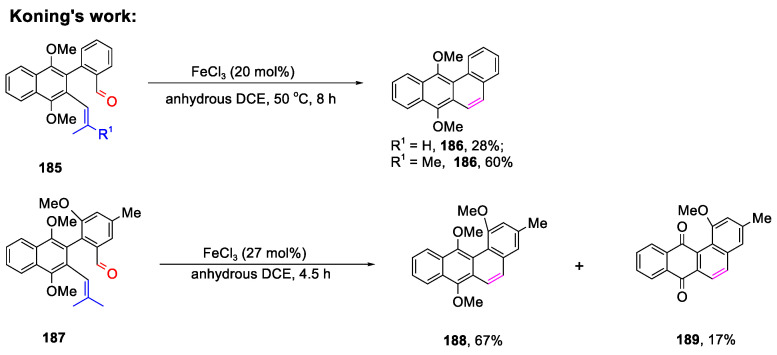
Synthesis of angucycline derivatives using carbonyl–olefin metathesis.

In 2017, Schindler and colleagues introduced a potent method for constructing polycyclic aromatic hydrocarbon **184** (Scheme 46) [106]. The starting material (**183**) underwent carbonyl–olefin metathesis via an oxetane intermediate, affording the corresponding metathesis product **184** with yields up to 99%, compatible with both ketones and aldehydes. Notably, Brønsted acids like HCl and *p*-TsOH could not facilitate this transformation. An alkene evaluation disclosed the following: (1) the *E*- and *Z*-isomer of olefin functional groups in **183** had no adverse effect on the reactivity; (2) benzaldehyde could not impede the reaction progress; (3) there was no reaction employing **183** featuring a terminal alkene as a substrate; and (4) nonstyrenyl substrate **183** could promote the competing carbonyl-ene reaction pathway, leading to a low yield of **184a**. In addition, various polycyclic skeletons, such as phenanthrene **184a** and benzothiophene **184h**, could be obtained. Interestingly, two carbonyl–olefin metathesis processes catalyzed by FeCl_3_ could proceed to offer the expected product, **184j**. Notably, only oxetane **184k** was isolated using the prenylated analog of **183** as a substrate. Later, angucycline **188** and tetrangulol skeleton **189** were furnished in yields of 60% and 67%, respectively, through this transformation, improving the catalytic loading to 20–27 mol% (Scheme 47) [107].

**Scheme 48:**
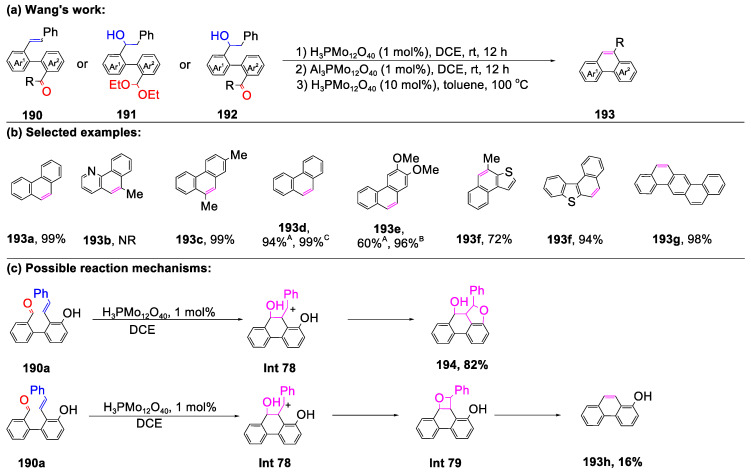
Brønsted acid-catalyzed carbonyl–olefin metathesis. A represents the yield obtained under condition 1); B represents the yield obtained under condition 2); C represents the yield obtained under condition 3).

Recently, a metathesis reaction involving ketone–olefin **190**, aldehyde–olefin **190**, acetal–alcohol **191**, and carbonyl–alcohol **192** was successfully catalyzed by the Brønsted acid H_3_PMo_12_O_40_ (Scheme 48) [108]. Both naphthyl-based and heteroaromatic ring-derived substrates were found to be compatible with this catalytic process. Nonetheless, benzo[*h*]quinoline scaffold **193b** could not be synthesized due to competitive binding with the pyridine skeleton, which hindered the reaction progression. Furthermore, the inherently sluggish reactivity of esters and amides (where R = MeO or NH_2_) resulted in the failure to generate the expected product under optimized reaction conditions. Intriguingly, when an *E*/*Z* mixture of olefin was used as the substrate, the corresponding product was successfully furnished. Additionally, both the double in situ metathesis of acetal–alcohol and a sequential dehydration/in situ metathesis of carbonyl–alcohols proceeded smoothly, affording the desired product with a good yield. Notably, the metathesis of carbonyl–alcohols did not proceed when a traditional Lewis acid was employed. These mechanistic studies indicated that the reaction proceeded via a stepwise mechanism (Scheme 48c), involving cation intermediate **Int 78**, which was stabilized by a vast anion cavity structure centered around a tetrahedral (PO_4_) unit, surrounded by four octahedral (MoO_6_) metal units.

### 4.2. The Formation of Polycyclic Compounds Catalyzed by Metal-Free Conditions

**Scheme 49:**
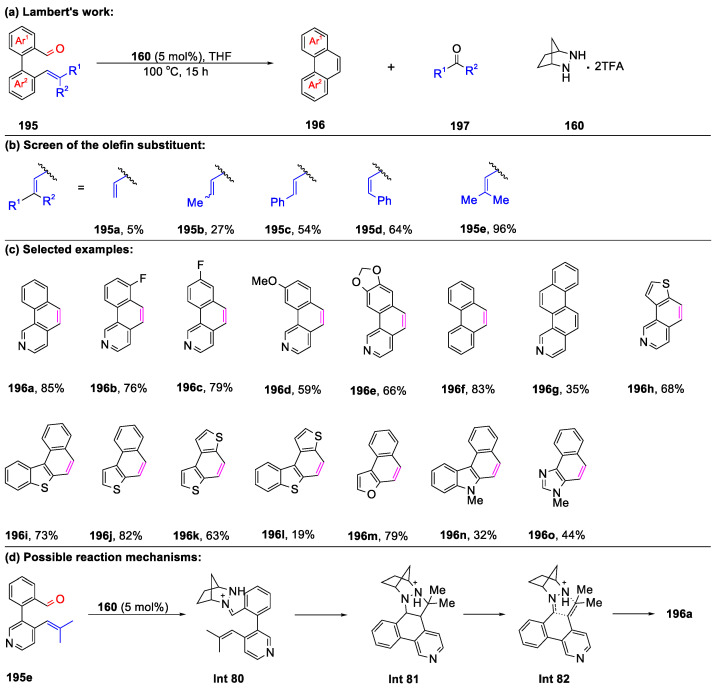
Hydrazine-catalyzed ring-closing carbonyl–olefin metathesis.

As a supplement to previous research, Lambert’s team achieved a remarkable breakthrough by catalyzing the ring-closing carbonyl–olefin metathesis of biaryl alkenyl aldehyde **195** using hydrazine as a catalyst. This process efficiently yielded polycyclic heteroaromatic compound **196**, with an excellent yield achieved using just 5 mol% of **160** (Scheme 49) [109]. A diverse range of anticipated products, including benzo[*h*]isoquinolines (**196a**–**196e**), phenanthrene (**196f**), thiophene-fused polycyclic heteroaromatics (**196h**–**196l**), naphthofuran (**196m**), benzocarbazole (**196n**), and naphthoimidazole (**196o**), were constructed in moderate yields. An olefin substitution screen revealed that 2-methyl-1-propenyl emerged as the optimal substituent for this transformation. Furthermore, DFT calculations were performed, elucidating the reaction mechanism. The condensation between the starting material (**195**) and [2.2.1]-hydrazinium **160** yielded the intermediate **Int 80**. Subsequently, an intramolecular cycloaddition between the olefin and the 1,3-dipole occurred, leading to the formation of **Int 81**. This intermediate then underwent a cycloreversion process via **Int 82**, ultimately producing the desired product, **196a**.

## 5. Conclusions

In summary, we provided an up-to-date review of the cyclization of carbonyl–olefin metathesis for the synthesis of monocyclic skeletons, bicyclic scaffolds, and polycyclic frameworks. These complicated frameworks were furnished through the fragmentation of oxetane, the cycloreversion of pyrazolidine formed by the [3+2]-cycloaddition of a carbonyl with hydrazine, and a metal alkylidene intermediate. Since the carbonyl–olefin metathesis transformation was disclosed in 1960, significant progress has been made within the last ten years. However, there remain many challenging tasks. Firstly, the functional group tolerance and broadening the substrate scope should be solved to enable efficient applications in the construction of complex molecules. Meanwhile, the protocol tolerating exceedingly Lewis basic sites and producing medium- and larger-sized rings should be prioritized for development. Moreover, the isomerization of newly formed olefins is commonly presented in current methods. In addition, enhancing the distinct reactivity mode of a carbonyl with alkene functionalities was a key branch in this field. Photocatalytic carbonyl–olefin metathesis is still in its infancy. Notably, the interrupted cycloreversion of oxetane has become a current frontier to afford heterocyclic and polycyclic compound skeletons.

## Data Availability

Not applicable.
